# Divergent immune microenvironments in two tumor nodules from a patient with mismatch repair-deficient prostate cancer

**DOI:** 10.1038/s41525-024-00392-1

**Published:** 2024-01-22

**Authors:** Hannah E. Bergom, Laura A. Sena, Abderrahman Day, Benjamin Miller, Carly D. Miller, John R. Lozada, Nicholas Zorko, Jinhua Wang, Eugene Shenderov, Francisco Pereira Lobo, Fernanda Caramella-Pereira, Luigi Marchionni, Charles G. Drake, Tamara Lotan, Angelo M. De Marzo, Justin Hwang, Emmanuel S. Antonarakis

**Affiliations:** 1 Department of Medicine, University of Minnesota-Twin Cities, Minneapolis, MN USA; 2 Division of Hematology, Oncology and Transplantation, University of Minnesota-Twin Cities, Minneapolis, MN USA; 3 Sidney Kimmel Comprehensive Cancer Center, Johns Hopkins, Baltimore, MD USA; 4 Institute for Health Informatics, University of Minnesota, Minneapolis, MN USA; 5 Masonic Cancer Center, University of Minnesota, Minneapolis, MN USA; 6 Department of Oncology, Johns Hopkins School of Medicine, Baltimore, MD USA; 7 The Bloomberg–Kimmel Institute for Cancer Immunotherapy, Johns Hopkins School of Medicine, Baltimore, MD USA; 8 Universidade Federal de Minas Gerais, Belo Horizonte, MG Brazil; 9 Pathology and Laboratory Medicine, Weill Cornell Medical College, New York, NY USA; 10 Present Address: Janssen Research and Development, LLC, Springhouse, PA USA

**Keywords:** Prostate cancer, Cancer microenvironment

## Abstract

Patients with prostate cancer (PC) generally do not respond favorably to immune checkpoint inhibitors, which may be due to a low abundance of tumor-infiltrating lymphocytes even when mutational load is high. Here, we identified a patient who presented with high-grade primary prostate cancer with two adjacent tumor nodules. While both nodules were mismatch repair-deficient (MMRd), exhibited pathogenic *MSH2* and *MSH6* alterations, had a high tumor mutational burden (TMB), and demonstrated high microsatellite instability (MSI), they had markedly distinct immune phenotypes. The first displayed a dense infiltrate of lymphocytes (“hot nodule”), while the second displayed significantly fewer infiltrating lymphocytes (“cold nodule”). Whole-exome DNA analysis found that both nodules shared many identical mutations, indicating that they were derived from a single clone. However, the cold nodule appeared to be sub-clonal relative to the hot nodule, suggesting divergent evolution of the cold nodule from the hot nodule. Whole-transcriptome RNA analysis found that the cold nodule demonstrated lower expression of genes related to antigen presentation (HLA) and, paradoxically, classical tumor immune tolerance markers such as PD-L1 (*CD274*) and CTLA-4. Immune cell deconvolution suggested that the hot nodule was enriched not only in CD8+ and CD4 + T lymphocytes, but also in M1 macrophages, activated NK cells, and γδ T cells compared to the cold nodule. This case highlights that MMRd/TMB-high PC can evolve to minimize an anti-tumor immune response, and nominates downregulation of antigen presentation machinery (HLA loss) as a potential mechanism of adaptive immune evasion in PC.

## Introduction

Prostate cancer (PC) is the most prevalent cancer in men and remains the second most common cause of cancer-related mortality^1^. While most deaths occur due to metastatic disease, treatment for localized PC is curative in many patients and the 5-year survival rate is greater than 95%^2^. In both early and advanced settings of PC, the androgen receptor (AR) remains a critical driver and therapeutic target^3–5^. Androgen deprivation therapies (ADT) and AR-targeted therapies (ART) are two therapeutic strategies that disrupt oncogenic signaling associated with the AR. While these therapies extend survival in patients with PC, resistance to ADTs and ARTs is inevitable. Several alternative targeted therapies are also being developed including those that target DNA damage repair (PARP inhibitors)^6–8^, PTEN loss (AKT inhibitors)^9^, and radiotherapies that target PSMA (Lu177-PSMA-617)^10^. In addition to these molecularly-targeted therapies, other strategies are leveraging a patient’s own immune system to treat their cancer. However, despite favorable outcomes in other cancer types, the use of immune checkpoint inhibitors (ICIs) and other immunotherapeutic strategies (including cell-based therapies and cancer vaccines) have proven less effective in PC^11^.

In broad oncology practice, clinical predictive biomarkers for ICI sensitivity include PD-L1 expression, TMB greater than 10 mutations per megabase (10 muts/Mb) and MSI-high status^12,13^. However, these markers are observed in only 3–5% of patients with PC^14^. The majority of PC tumors are regarded as immunologically cold, meaning that they display a low abundance of tumor infiltrating T-cells. This is thought to be one reason that patients with PC have poor response towards current immune-directed therapeutic strategies^15,16^. Due to the very small population of patients with PC who successfully mount anti-tumor immunity, it has been a challenge to identify key features that enable or suppress immune responses toward PC.

Here, we have identified a unique case of a patient with high-risk localized PC with two adjacent tumor nodules displaying distinctly divergent infiltrating immune-cell phenotypes. Despite both nodules being TMB-high and MSI-high, one nodule demonstrated a markedly higher density of tumor-infiltrating T cells. We interrogated these two tumor nodules to discern molecular differences between their exomes, transcriptomes, and tumor microenvironments to identify possible mechanisms that regulate immune-cell interactions and immune evasion in PC.

## Results

### Case report

**Fig. 1:**
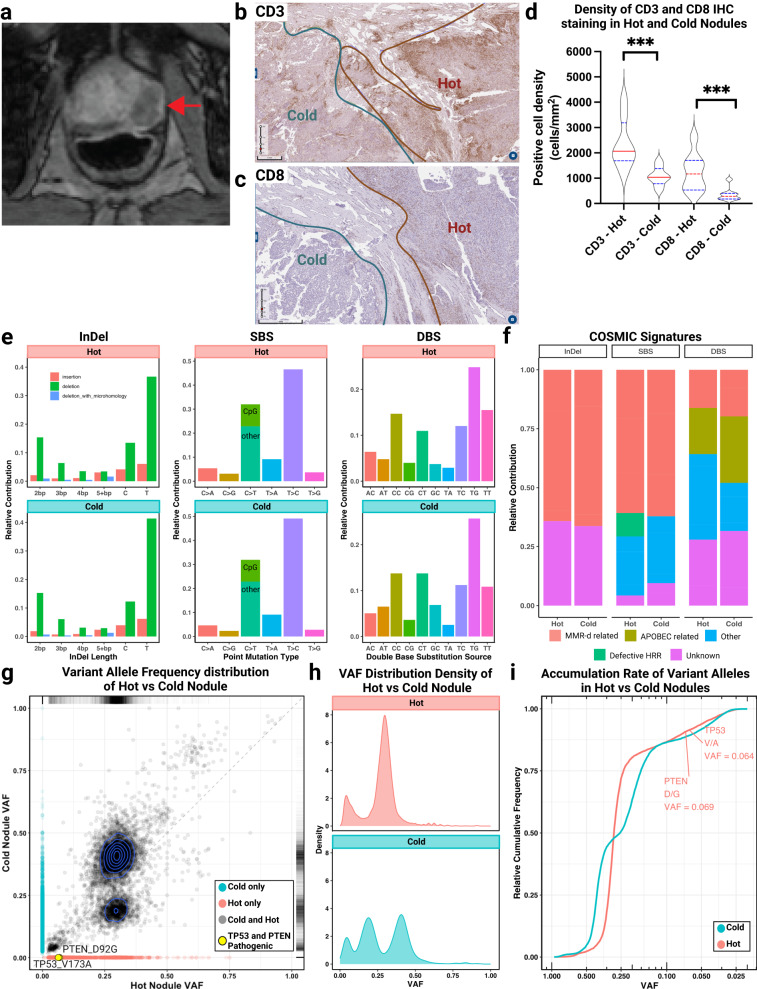
A “hot” and “cold” tumor nodule in the prostate gland, with separate whole-exome analysis of each nodule. **a** T2-weighted axial MRI image of the prostate shows entire left peripheral zone is replaced by tumor bulging into the capsule (arrow). Immunohistochemistry (IHC) against CD3 (**b**) and CD8 (**c**) on the prostate tumor tissue. Positive cells for CD3 or CD8 stain brown on the image. **d** CD3 and CD8 IHC staining were quantified based on the density of positive cells per mm^2^ based on counts on 8 independent regions in each nodule. **e** Patterns of insertion/deletions, single base substitutions, and double base substitutions are analyzed and depicted for the hot and cold tumor nodules, based on COSMIC analysis. **f** The relative contributions of genomic alterations scored based on COSMIC signatures in the two nodules. **g** Scatter plot depictions of all detected variants are shown between both nodules. Variants exclusive to the hot nodule are shown in pink, exclusive to the cold nodule are in teal, and shared between both in gray, pathogenic variants of interest are highlighted in yellow (*TP53* and *PTEN*). Two rare variants in the hot nodule (*TP53* and *PTEN*) are depicted. Relative density measurements are added to in which the blue circles reflect the distribution of the variants within the clusters that have high density. **h** A depiction of the clone numbers in the hot and cold nodules. **i** A pseudo-time analysis of the hot (pink) and cold (blue) nodules depicts variant accumulation rates and when the *PTEN* and *TP53* variants were accrued. Pseudo-times (x-axis) are plotted relative to the earliest (VAF = 1) and latest (VAF = 0) possible events.

A 61-year-old man was found to have a prostate-specific antigen (PSA) level of 10.4 ng/mL and a palpable prostate nodule that was clinical stage cT2b. A prostatic biopsy revealed adenocarcinoma in 5 of 12 cores, with Gleason grade 5 + 5 = 10. Computed tomography of the chest, abdomen, and pelvis and bone scintigraphy were negative for local or distant metastases. Magnetic resonance imaging of the prostate gland showed the entire left peripheral zone occupied by tumor with central necrosis, which deformed the capsule and abutted the left neurovascular bundle without frank invasion (Fig. 1a). Given the diagnosis of high-risk localized PC, the patient elected to enroll in a clinical trial of neoadjuvant treatment prior to radical prostatectomy (RP). The trial randomized patients with high-risk PC to treatment with androgen-deprivation therapy (ADT; degarelix 240 mg subcutaneously) given two weeks prior to RP (Cohort A) or to cyclophosphamide (200 mg/m2 intravenously) and a Granulocyte-macrophage colony-stimulating factor (GM-CSF)-secreting allogeneic cellular vaccine (GVAX; composed of 2.5 × 10^8^ PC3 cells and 1.6 × 10^8^ LNCaP cells, injected intradermally) plus ADT given two weeks prior to RP (Cohort B). The main objective of that trial was to analyze CD8 + T cell density in the tumor obtained at RP (NCT01696877)^17^. This patient was randomized to cohort B and was treated with cyclophosphamide/GVAX plus ADT followed by RP. His PSA level became undetectable post-operatively and remains undetectable at the time of writing, greater than 7 years after prostatectomy. This patient has achieved a 7-year cancer-free interval thus far despite having both hot and cold nodules present in his prostate. This suggests that even the cold nodule exhibited a favorable response to combination therapy consisting of RP, ADT and GVAX vaccination, likely due to the fact that this nodule also showed MMR deficiency and hypermutation.

On pathologic examination of the patient’s RP specimen (Supplementary Fig. 1), there was invasive high-grade prostate adenocarcinoma that occupied relatively large areas of the peripheral zone of the prostate gland. Upon medium-power view, two nearby yet distinct appearing nodules were present in the left peripheral zone that were separated by an intervening stromal component (Supplementary Fig. 1A). Each nodule displayed high-grade prostate adenocarcinoma (Gleason 4 + 5 = 9), but one showed a higher density of infiltrating mononuclear inflammatory cells consistent with lymphocytes (termed the “hot” nodule) compared with the other nodule (termed the “cold” nodule) (Fig. 1b, c). By single-plex chromogenic immunohistochemistry, the hot nodule showed a much greater density of CD3+ and CD8+ tumor-infiltrating T cells (TILs) than the cold nodule (Fig. 1b, c and Supplementary Fig. 1D–I). The increased number of CD3 and CD8 cells staining in the hot nodule was supported by our quantification in which we determined the density of positive cells per mm^2^ (Fig. 1d). In addition to there being a relatively high density of CD8+ and CD3 + T cells in the tumor interior, the hot nodule also contained dense collections of these cells in a band-like pattern toward the periphery of the tumor (Fig. 1b and Supplementary Fig. 1D)—a highly unusual feature for PC. Given that TIL density is an established biomarker of immunologic recognition and anti-tumor^18^, we sought to characterize molecular differences between these nodules that could inform mechanisms of immune stimulation in the hot nodule or immune evasion in the cold nodule.

### Genomic analysis

With such striking differences observed in immune-cell densities on histology, we anticipated divergent (or separate) genomic profiles between these nodules. However, genomic sequencing of bulk tumor tissue using Personal Genome Diagnostics (PGDx) showed that both the hot and cold nodules demonstrated evidence of MMRd and extremely high TMB. As compared with aggregated data from PC which has a median TMB of 2–3 muts/Mb, in the hot nodule the TMB was 131 muts/Mb, the frameshift burden was 12 frameshifts/Mb, and the frameshift proportion was high at 9%. Similarly, in the cold nodule the TMB was 164 muts/Mb, the frameshift burden was 15 frameshifts/Mb, and the frameshift proportion was 9%. In a comparative analysis relative to all other cancer types in The Cancer Genome Atlas (TCGA) dataset, these nodules exhibited the second and third highest TMB relative to all other primary PC tumors (*n* = 495) and the second and sixth highest TMB relative to all other patients (*n* = 468) even amongst mutation-high cancers such as melanomas (Supplementary Figure 2). Furthermore, both nodules harbored inactivating mutations in *MSH2* and *MSH6* (*MSH2* p.E809*, *MSH6* p.F1104Lfs*11) with loss of protein expression by immunohistochemistry, confirming MMRd status, and were MSI-high. Germline genetic testing was unremarkable, confirming that these mutations were acquired somatic events. Both nodules also had *POLE* mutations (p.Y2003C, p.A782V, and p.D756G) although none were in the exonuclease domain. Additional mutations identified and annotated with Ensembl’s variant effect predictor (VEP)^19^ are listed in Supplementary Table 1. Due to the unexpected finding of MMRd and high-TMB in both tumor nodules, we sought to further comprehensively characterize additional molecular features that may explain the distinct TMEs seen in the two nodules.

To this end, we first examined COSMIC mutational signatures in the two tumors including insertion and deletions (InDel), single-base substitutions, and double-base substitutions between the hot and cold nodules (Fig. 1e)^20^. In this regard, the two nodules showed a high degree of similarity between their mutational processes at the nucleotide level, providing strong evidence that they were clonally related. We then examined the relative contribution of each signature type in the two nodules (Fig. 1f); again, both nodules showed predominantly MMRd-related mutational patterns. Interestingly, the hot nodule displayed a small relative contribution of defective homologous recombination repair (HRR) that was not observed in the cold nodule, even though comparable variant allele frequencies (VAFs) of *BRCA1/2* alterations were observed and none of them were pathogenic. We next sought to examine the landscape of VAFs in both nodules; here, we found unique patterns in the hot nodule. Specifically, the hot nodule exclusively showed unique pathogenic mutations in *TP53* and *PTEN* (Fig. 1g), although both of these mutations were of low variant allele frequency. Further, the hot nodule was composed of a single tumor clone while the cold nodule was composed of two clones (Fig. 1h). We next modeled the order in which mutations accumulated through a pseudo-time analysis using the VAFs of all detectable gene alterations. Based on this approach, the cold nodule initially accumulated mutations quicker and at an earlier time point compared to the hot nodule (Fig. 1l). At a later pseudo-time, the cold nodule exhibited a similar overall rate of variant accumulation as the hot but never acquired the unique pathogenic *TP53* and *PTEN* mutations. While the overarching mutational processes appeared very similar, these nuanced genomics analyses indicated that these tumors exhibited distinct rates of VAF accumulation and harbored certain unique mutations.

### Transcriptomic analysis

**Fig. 2:**
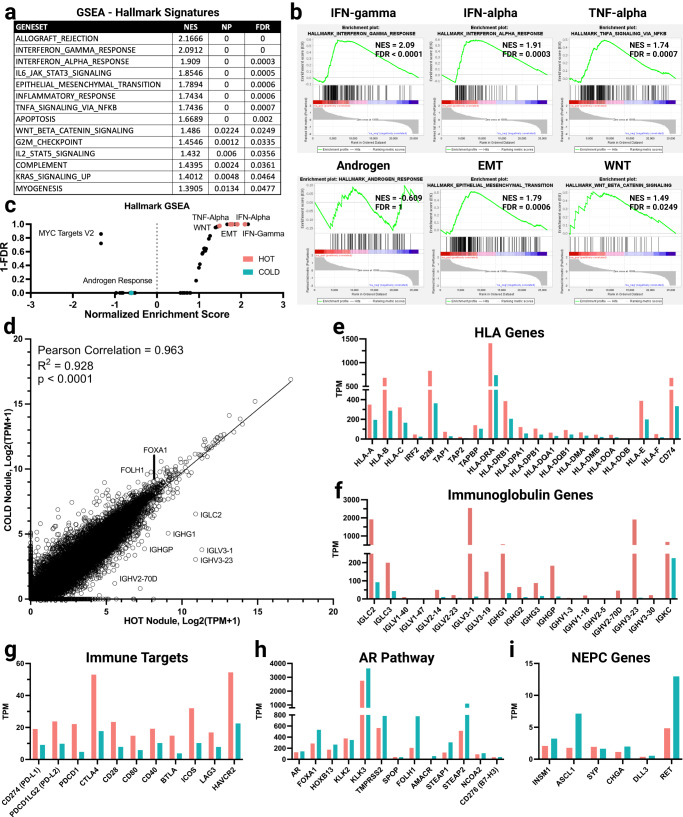
Transcriptome analyses of the hot and cold tumor nodules. **a** Gene set enrichment analysis (GSEA) was performed on the transcriptomes of the hot and cold nodules where the pathways significantly enriched in the hot nodule are highlighted (NES – normalized Enrichment score, NP – nominal p-value, FDR – false discovery rate). **b** GSEA results are depicted for immune regulatory, oncogenic, and prostate cancer-associated signaling pathways. Results reflect relative enrichment in the hot (positive NES) or cold (negative NES) nodules. Net enrichment score (NES) and false discovery rate (FDR) are presented for each signature. **c** The NES and 1-FDR for the GSEA results are depicted in a snake plot for all Hallmark GSEA signatures where specific signatures are highlighted that are enriched in the hot (pink) or in the cold (blue) nodule. **d** The overall similarity of the transcriptomes in the hot and cold nodule are evaluated via a Pearson correlation (0.963) and the statistical significance is reported (*p* value < 0.0001). The expression level (TPM) for individual genes is shown in the hot (pink) and cold (blue) nodule as follows: **e** HLA-related genes. **f** Immunoglobulin genes. **g** Immune regulatory genes of which many are targets for immunotherapy. **h** AR-related genes. **i** Neuroendocrine PC (NEPC) genes.

We next sought to examine the whole transcriptomes (Fig. 2) of the hot and cold nodules, to interrogate potential differences in specific genes or overarching biological processes. We performed gene set enrichment analysis (GSEA) of the 50 hallmark signatures^21,22^, and expectedly found that the hallmark signatures enriched in the hot nodule were primarily related to immune-regulatory signaling pathways such as IFN-γ/α and TNF-α (Fig. 2a, b, c). Gene expression and genomic alteration status of each gene in the signatures are included as supplementary data (Supplementary Tables 4, 5). Otherwise, based on a Pearson correlation of relative transcript abundance, these tumors displayed strikingly similar overall transcriptomes (R^2^ = 0.928, *p* value < 0.0001) (Fig. 2d). Yet the hot nodule was enriched for a variety of immunoglobulin genes, including *IGLV3-1* and *IGHV3-23* among others (Fig. 2d, f). Interestingly, the hot nodule was also enriched in signatures associated with therapy resistance such as epithelial-to-mesenchymal transition (EMT)^23^ and WNT signaling^24^. While androgen signaling is thought to negatively impact the activity of immune cells^25,26^, the hallmark androgen response signature was not enriched in either the hot or cold nodule, suggesting that both had similar expression of *AR*-related genes (Fig. 2c, d, h). Despite this limited overall difference in the signaling pathways, individual AR-related and NEPC genes were all generally upregulated in the cold tumor (Fig. 2h, l).

When examining the expression of specific individual genes, the hot nodule displayed increased expression of many HLA^27^ genes including *HLA-B*, *B2M*, *HLA-DRA* and *CD74* compared to the cold nodule (Fig. 2e). When we examined epigenetic regulators that regulate HLA expression^28^, the hot nodule contained a unique *DNMT3B* variant while the cold nodule had a unique *DNMT3AP1* variant. Based on gene expression patterns, *TAP1*, *TAP2*, and *B2M* were all decreased in the cold nodule compared to the hot nodule. Similarly, the hot nodule was enriched in many immunoglobulin genes, which may be due to differences in relative abundance of B-cells and plasma cells. However, differences between the nodules are not robust when examined by CIBERSORT or IHC and therefore this does not directly explain the enrichment of immunoglobulin genes in the hot nodule (Fig. 2f, Supplementary Fig. 3). The hot nodule also had increased levels of ICI target genes such as *PD-1, PD-L1* and *CTLA4*. (Fig. 2g). While multiple immune-related genes had seemingly distinct expression patterns, there were limited differences when we examined critical regulators of AR signaling such as *FOXA1, HOXB13*^29,30^, and *KLK2/3*^31,32^ (Fig. 2h) or neuroendocrine PC regulatory genes such as *INSM1* and *ASCL1* (Fig. 2i). Altogether, these nuanced transcriptional analyses uncovered key transcriptional differences (characterized by loss of HLA gene expression and reduced immunoglobulin gene expression in the cold nodule) despite a seemingly similar broad transcriptional profile in the two nodules.

### Immune cell deconvolution

**Fig. 3:**
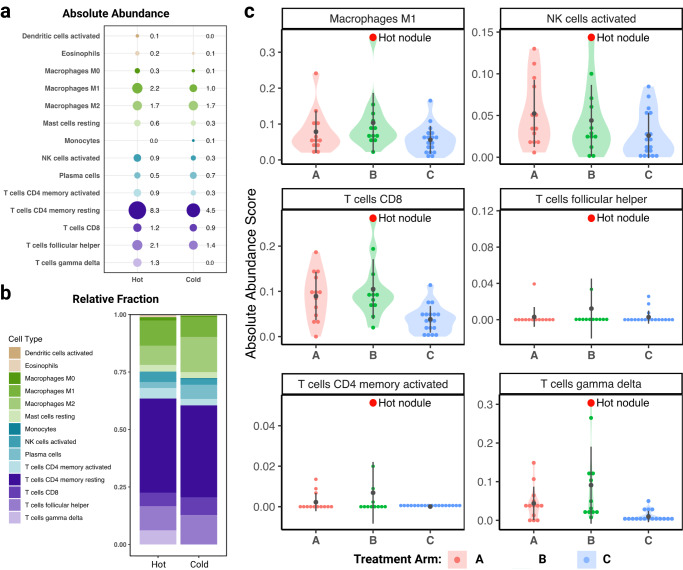
Immune cell landscape of the hot and cold tumor nodules. **a** CIBERSORT was utilized to infer the absolute abundance of immune cell subsets in the hot and cold tumors from WTS data. **b** CIBERSORT was utilized to infer the relative fraction of immune cells is presented by cell type in the hot vs cold nodules from WTS data. **c** NanoString data was used to compute the absolute abundance scores for several immune cell subtypes in the hot nodule (red). These scores were compared to all other patients in the original GVAX clinical study. Each treatment arm is depicted by a different color (A, pink – ADT alone; B, green – cyclophosphamide/GVAX and ADT; C, blue – untreated control group).

We next sought to comprehensively interrogate the TME in the two nodules, through inferential analysis via bulk RNA sequencing data. When evaluating projected abundance and relative fraction of immune cells using CIBERSORT^33,34^, the hot nodule exhibited higher levels of CD8+ and CD4 + T lymphocytes, as well as more M1 macrophages, activated natural killer (NK) cells, and gamma delta (γδ) T cells compared to the cold nodule (Fig. 3a, b). Other immune cell subtypes from CIBERSORT are summarized in Supplementary Tables 2 and 3. We next sought to explore if the hot nodule had differential immune-cell fractions when compared to other patients in the original GVAX clinical trial^17^. Using Nanostring IO360 gene expression data from a selective panel of immune-related genes (previously generated^17^), the absolute abundance of immune-cell subtypes was inferred using CIBERSORT^33,34^ and compared between patients enrolled on the trial (*n* = 43). To this end, the hot nodule from our patient displayed higher levels of many immune-cell subtypes relative to all other patients in the trial regardless of the treatment arm (Fig. 3c). Of note, Nanostring IO360 gene expression data from the cold nodule were not previously generated and could not be reported. Taken together, these immune-cell deconvolution analyses indicated that the hot nodule displayed a unique TME compared to all other PC patients in the GVAX clinical trial across all three arms.

## Discussion

TMB-high and MSI-high status in tumors are associated with increased immunogenicity, immune recognition, and elimination of tumor antigens by the immune system. Due to these factors, TMB and MSI status are currently used as clinical biomarkers to deploy ICI treatments in a histology-agnostic fashion^35,36^. Our study demonstrates that additional features may regulate the TME, and further investigations are required to identify mechanisms that drive immune-cell interaction in the 3–5% of PC tumors that are TMB-high and/or MSI-high. As indicated by two adjacent tumor nodules in this PC patient, the hot nodule had higher expression of HLA and immunoglobulin genes compared to the cold nodule. Or perhaps, viewed alternatively, the cold nodule lost HLA and immunoglobulin gene expression as a means of adaptive immune resistance. An alternative hypothesis is that the lower expression of HLA and immunoglobulin genes could have been the result of a sparser immune-cell infiltrate in the cold nodule due to the bulk RNA sequencing methodology used (rather than single-cell RNA sequencing). Interestingly, some recent studies have indicated that tumors can decrease expression of antigen-presentation genes in order to evade immune-cell surveillance^27,37^. As another possibility, each nodule also harbored alterations in regulatory genes and epigenetic factors that regulate immune surveillance^28,38^. This may have contributed to the reduced immune surveillance functions in the cold nodule. We also found that both nodules exhibited largely similar mutational processes, with the exception of a component of HRR, but had distinct numbers of subclones and VAF accumulation rates.

Of note, our GSEA analysis indicated that the hot nodule was interestingly enriched in EMT and WNT signaling, two pathways typically associated with the immune evasion^39,40^. Our study implicates other pathways may have led to the distinct immune cell phenotypes in these nodules. With respect to ICI implications, our findings indicate that clinical-grade molecular tests should consider reporting immune-regulatory signatures and relative expression levels of HLA and immunoglobulin genes in addition to TMB and MSI status.

Currently, the low response rates of heterogeneous solid tumors, including PC^41^, to single-agent immunotherapies has led to active investigation of combination strategies. Our patient was enrolled in a trial in which ADT was combined with a cellular PC vaccine, GVAX^17^. Interestingly, both the hot and cold nodules shared variants with GVAX based on the variant profiles of PC3 and LNCaP cells found in the DepMap database. While the hot nodule overall shared less variants with these cell lines (Supplementary Table 6), it remains possible that specific highly-immunogenic variant(s) produced antigens that mediated the inflamed response. Previous work has demonstrated that androgens may suppress T-cell function^26^, and that inhibition of AR (via ADT and/or ART) may alter the TME by enhancing T-cell function^25^ The rationale of the GVAX trial was that ADT would complement the vaccine to promote infiltration of pro-inflammatory immune cells and ultimately CD8 + T-cells into tumors^42^. In this patient, both nodules underwent the same systemic therapy, which indicates that mechanisms other than ADT drove the significant differences in the TMEs. Stromal cells may also contribute to the divergent immune phenotypes^43^. The cold nodule was visually associated with greater proportions of stromal cells. However, functional mapping with single cell resolution (which was not possible here) is required to determine if specific stromal cells may have contributed to the differences in immune cell infiltration. A more refined understanding, which will require more patient studies, is necessary to identify the recurrent mechanisms that alter the TME in PC patients, which may impact the deployment of immune checkpoint blockade, cell-based therapies, and vaccine therapies in solid tumors.

We also demonstrate here that a prostate tumor may interact with many forms of immune cells in its microenvironment. Outside of regulatory and helper T-cells, we inferred enrichment of γδ T-cells, M1 macrophages, and activated NK cells in the hot nodule relative to the cold nodule. Studies have shown that NK^44^ or γδ T-cells^45,46^ are able to invade and kill tumors. As compared to T-cells, which have been less effective in driving tumor response in solid tumors, further research should measure the relative infiltration rates of NK and/or γδ T cells in the tumor microenvironment, which may allow us to consider such cell-based therapies for treating prostate tumors^47^.

There are technical and statistical limitations based on this case study of two nodules. We have made descriptive observations based on thresholds that would typically be considered notable in clinical reports or larger patient cohorts. However, validating function and causality would still require further modeling of the patients’ tumors. To generalize our key observations, we would also require validation of key findings in greater cohorts of dMMR tumors.

In conclusion, our case study highlights that complex genomic and transcriptomic alterations can regulate the TME in TMB-high and MSI-high PC tumors. Therefore, in addition to examining mutational burdens and signatures, it is of relevance to interrogate additional immune-regulatory and clonal processes, as well as to consider the types of immune cells that are associated with the tumor and its microenvironment. Finally, the observation of HLA loss in the context of MMR-deficient cancers observed here (and reported elsewhere)^48^ may have broader relevance as an immune evasion mechanism in other tumor contexts. In this patient with an ultra-high TMB in both the hot and cold nodules, it is tempting to speculate that the immunogenic effect of accumulating mutations may have been counterbalanced by transcriptional events that dampened immunoreactivity.

## Methods

### Patient approval

The patient provided written consent for inclusion in the clinical trial, for further genomic analysis of their tumor as presented herein, and to publish the findings.

### Ethics approval

This study has complied with all relevant ethical regulations including the Declaration of Helsinki. IRB approval was obtained for the data related to the initial clinical trial and further details can be found in the original manuscript publication^17^. Patient consent was acquired for the additional sequencing on the two tumor nodules and the analyses included in this manuscript. All other data included in this study was derived from public resources and these resources are provided in the manuscript. Ethical approval from the source datasets included can be found in the respective sources.

### Sample preparation and sequencing

Sample preparation, library construction, exome capture, and next generation sequencing of FFPE tumor and normal saliva samples were performed at Personal Genome Diagnostics (Baltimore, MD). Total DNA and RNA samples were isolated from patient FFPE tumor tissue specimens and sequencing was performed using the HiSeq2500 system with 100 base pair reads at Personal Genome Diagnostics (Baltimore, MD). Further details of sample preparation procedures and sequencing parameters can be found in a prior study by Jones, S. et al.^49^.

### Whole Exome Sequencing (WES) Data seq processing

Raw fastq files were obtained and mapped to the GRCh38 reference genome using BWA MEM^50^. Picard tools were used for BAM sorting and marking duplicate reads. Base quality score recalibration was done using GATK^51^. Mutect2 was used for somatic variant calling, with a patient matched normal saliva sample used as input alongside the tumor samples. Tumor VCF files were annotated with Ensembl VEP^19^. These WES data preprocessing steps were conducted using Minnesota Supercomputing Institute High Performance Computing resources on a CentOS Linux environment. Mutational signatures were analyzed using the MutationalPatterns^20^ package in R, with which COSMIC mutational signatures were used to infer DNA damage signatures^52^. The maftools package in R was used to generate and plot annotated VAF results. The tcgaCompare function from the maftools R package was used to compute the tumor mutational burden (TMB) of the two prostate nodule samples and compare them to 33 different TCGA datasets. Details regarding the TCGA studies included in the function and in our analysis can be found here: https://github.com/PoisonAlien/TCGAmutations.

Microsatellite instability (MSI) status and tumor mutational burden (TMB) were defined as previously described^53^. Frameshift mutation burden was defined as the number of insertion/deletion frameshift mutations per Megabase of DNA (muts/Mb), as previously described^54^. Frameshift mutation proportion was defined as the proportion of frameshift mutations relative to all nonsynonymous sequence alterations per Megabase of DNA (muts/Mb), as previously described^54^.

### Whole transcriptome sequencing (WTS) data processing

Transcriptome sequence data processing and analysis were performed using pipelines at the Minnesota Supercomputing Institute and University of Minnesota Informatics Institute (UMII) at the University of Minnesota. Raw reads were trimmed, aligned to the GRCh38 human genome, and gene-level read counts were generated using the CHURP pipeline^55^. All downstream gene expression analyses and visualizations were conducted using R (4.2.1), RStudio (2022.07.2 + 576) and GraphPad Prism 9. Transcripts per million (TPM) were calculated from raw counts for use in all downstream transcriptomic analyses and visualizations.

### Nanostring data processing

Normalized count gene expression data from the Nanostring IO360 immune gene panel (https://nanostring.com/products/ncounter-assays-panels/oncology/pancancer-immune-profiling/) consisting of 730 genes was obtained for 43 patients from the GVAX trial, including the hot tumor nodule from the patient of interest (sample 15-21233).

### Gene set enrichment analysis (GSEA)

The differences in transcripts per million for each gene was calculated and divided by the mean expression values in T1 and T2. These values were used to generate a ranked profile in order to conduct the subsequent pre-ranked GSEA using Hallmark Signatures^21^.

### CIBERSORT analysis

CIBERSORTx^33,34^ was used to estimate immune cell fractions and abundances. The LM22 microarray dataset, consisting of 547 genes that distinguish 22 mature human hematopoietic populations, was used as the signature matrix input for the imputation of immune cell fractions in the tumor samples. Normalized whole-transcriptome data were used to compare immune cell fractions and abundance of the two prostate nodules. Nanostring data were used to compare immune cell abundance across 43 patients in the GVAX trial.

#### Statistics and reproducibility

Pairwise gene correlation coefficients (Pearson’s), *p* values, and adjusted *p* value statistics were calculated using R 4.2.2 (R Core Team; 2022), the stats (v4.2.2; R Core Team; 2022), the Tidyverse (v1.3.2; Wickham; 2019), and the Hmsic (v4.7-2; Harrell Jr F; 2022) packages.

### IHC and image analysis for CD8 Cell IHC density measurements

IHC staining for CD8 was performed previously as a single-plex assay with DAB as chromogen (PMID: 32173650). The whole slide image was uploaded to HALO 3.6.4134.137 (Indica Labs). Each region (hot nodule and cold nodule) was separately annotated. The HALO random forest algorithm was used to train a classifier to detect total tissue area in the annotated region, which excluded lumens and bare glass areas. The Cytonuclear 2.0.9 algorithm was used to segment the cells and analyze the CD8 positively stained cells. The cell segmentation was based on hematoxylin staining and the following parameters: nuclear contrast threshold, minimal nuclear optical density, nuclear size and nuclear segmentation aggressiveness.

### Reporting summary

Further information on research design is available in the Nature Research Reporting Summary linked to this article.

### Supplementary information

Supplementary Information

Supplementary Tables 1-6

REPORTING SUMMARY

## Data Availability

Both the hot and the cold nodules tumor from this patient underwent bulk whole-exome DNA sequencing (WES) and bulk whole-transcriptome sequencing (WTS) at PGDx (Baltimore, MD). The data corresponding to this study is now available at the SRA (BioProject Accession number PRJNA1032970) and GEO (Accession number GSE246684). The additional Nanostring data from the original clinical trial that was analyzed during this study is included in a published article and its supplementary files (10.1158/1078-0432.CCR-19-3372)^17^.

## Code availability

The basic operational source code has been deposited into the following Zenodo Digital Repository (10.5281/zenodo.10206909). Additional requests regarding codes and scripts can be addressed to the corresponding author.
