# Supplementary material for: Divergent immune microenvironments in two tumor nodules from a patient with mismatch repair-deficient prostate cancer
Source: NPJ Genom Med. 2024 Jan 22;9:7. doi: 10.1038/s41525-024-00392-1 (PMC10803790; doi:10.1038/s41525-024-00392-1)
Supplement: Supplementary file 1 — Supplementary Information [file 41525_2024_392_MOESM1_ESM.docx]

**Supplementary Figures and Figure Legends.**

**
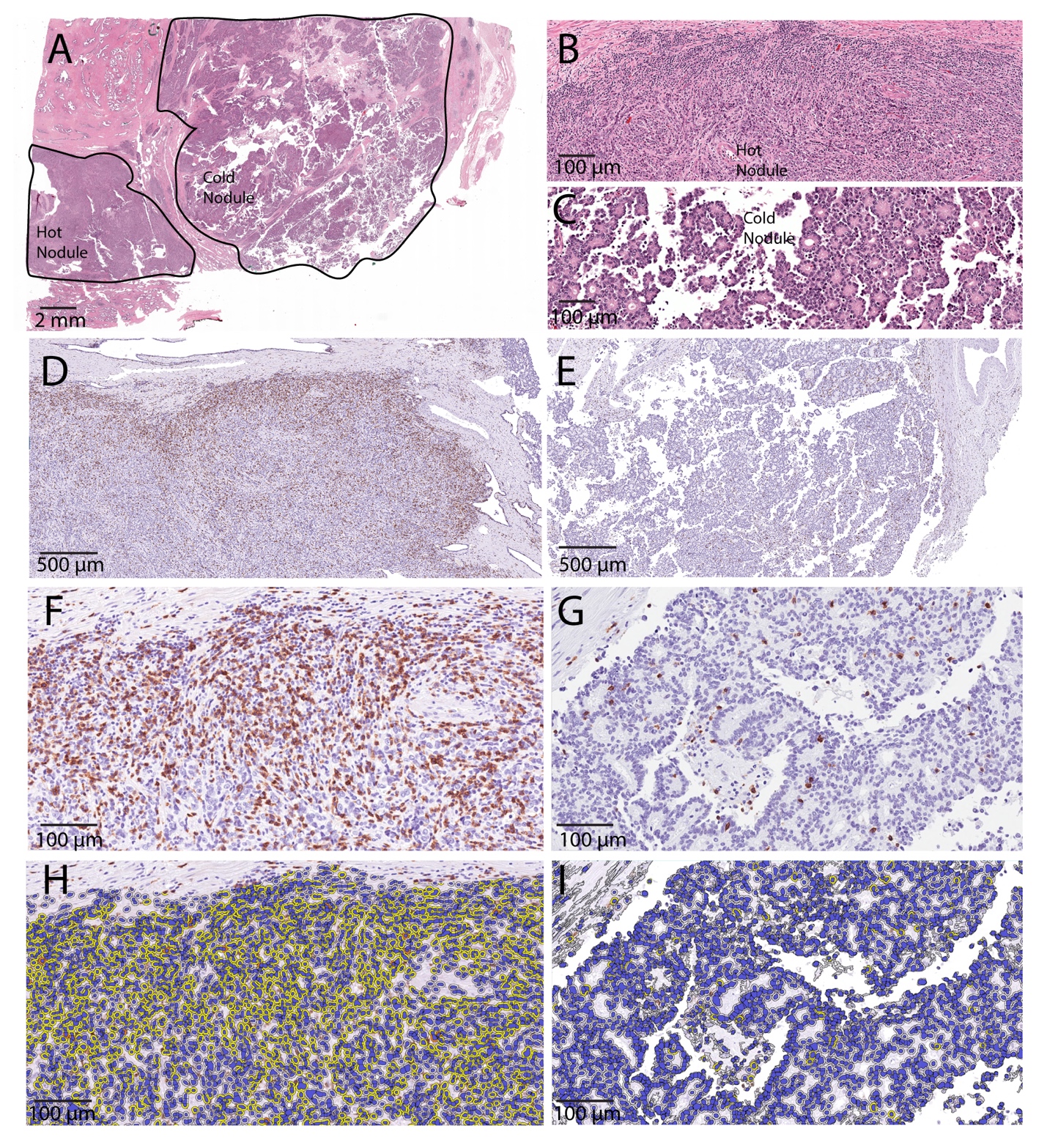
**

**Supplementary Figure 1. H&E and IHC staining of two tumor nodules reveals divergent infiltrating immune-cell phenotypes.**

**A**. Low power view of H&E image showing both hot and cold nodules. **B** **and** **C.** Higher power views of H&E images from the hot (**B**) and cold (**C**) nodules showing much greater cellularity in the hot nodule, consisting of higher-grade carcinoma cells and increased numbers of infiltrating mononuclear cells. **D**. CD8 staining of low power view of hot nodule showing brown staining CD8 cells that are quite abundant and are present in a band-like pattern on the periphery of the nodule. **E**. CD8 staining in the cold nodule showing a much lower number of T cells. **F and G**. Higher power views of **D** and **E**. **H and I** The cellular segmentation analysis for the hot (**H)** and cold (**I**) nodules of CD8 cells (positive staining cells show a yellow or orange outline) and total cells (negative staining cells show a gray outline). Nuclei are shown in blue.

**
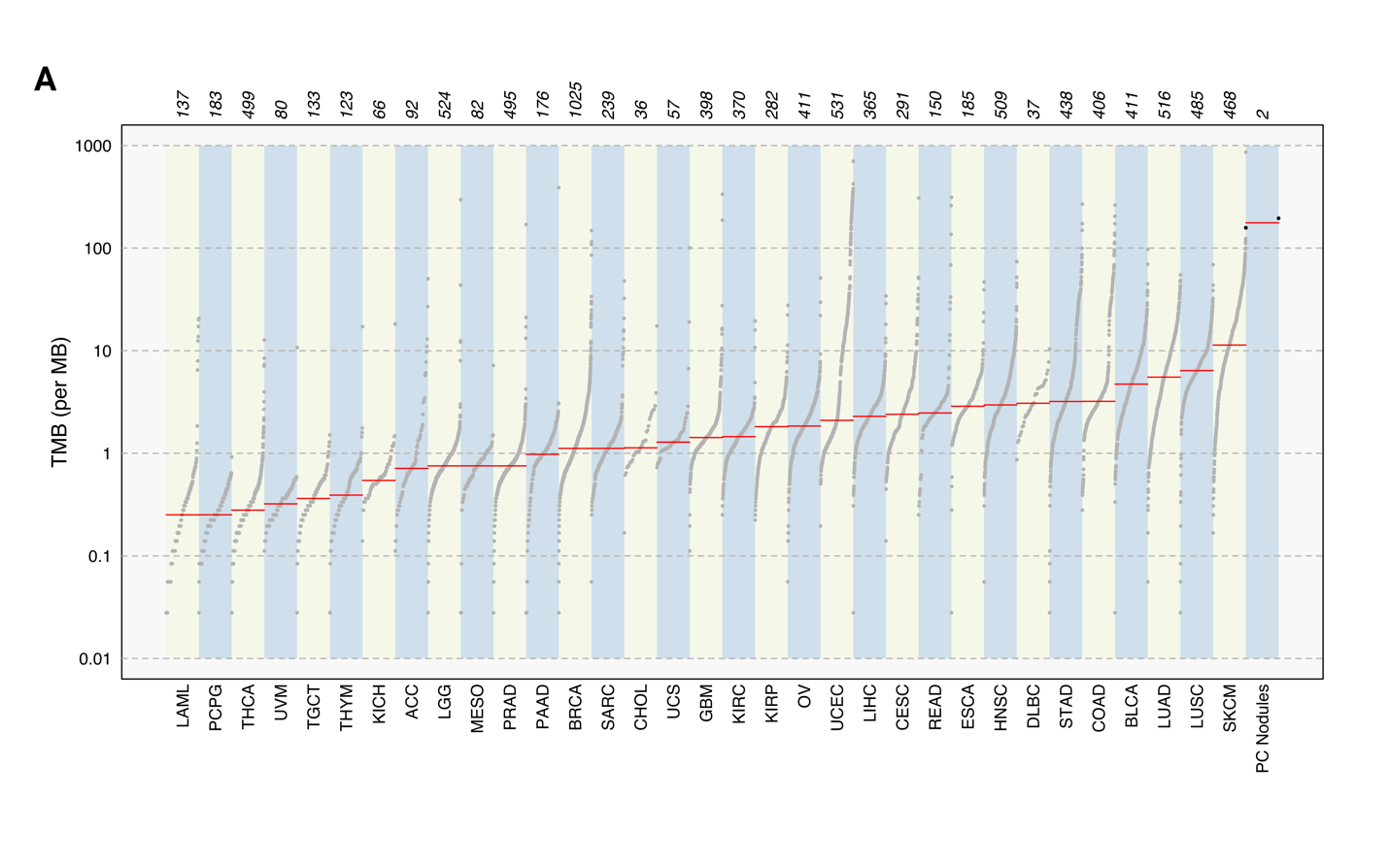
**

**Supplementary Figure 2. Pan-cancer comparison of TMB between TCGA and this patient.**

**A**. TMB was ranked from low to high and depicted via a snake plot across all patients within a cancer type in TCGA (grey) and in the hot and cold nodules (black) from our patient. The median TMB for each cancer type is highlighted in red. The number of patients (n) in each cancer type is indicated on the upper x-axis.

**Supplementary Figure 3**. **IHC staining for B-cells and Plasma cells in Hot and Cold nodules.** CD79a (B cells) and CD138 (plasma cells) IHC staining of the Hot (Left) and Cold (Right) nodule. The small arrows indicate immune cells, whereas the larger arrows indicate stained epithelial tissue.

**Supplementary Tables.**

**Supplementary Table 1**. Pathogenic mutations of clinical significance in hot and cold tumor nodules.

**Supplementary Table 2**. CIBERSORT relative fractions of immune cells in hot and cold tumor nodules.

**Supplementary Table 3**. CIBERSORT absolute abundance of immune cells in hot and cold tumor nodules.

**Supplementary Table 4** - Gene Expression (TPM) values in the hot and cold nodule, organized by hallmark signature.

**Supplementary Table 5 –** All detected genomic variants in the hot and cold nodule, organized by hallmark signature.

**Supplementary Table 6** - Overlapping genomic variants between the two nodules and GVAX components.
